# Picturing Illness, Making Meaning: A Virtual Photovoice Study of Systemic Lupus Erythematosus Narratives Among Chinese Women

**DOI:** 10.3390/bs16071197

**Published:** 2026-07-16

**Authors:** Ning Xu, Hongzhe Xiang, Yongkang Hou

**Affiliations:** 1 Faculty of Humanities and Arts, Macau University of Science and Technology, Macao SAR, China; 2 School of Journalism and Communication, Tsinghua University, Beijing 100084, China

**Keywords:** systemic lupus erythematosus, chronic care, patient-centered care, self-management, Photovoice, invisible illness, China

## Abstract

**Background/Objectives**: Systemic lupus erythematosus (SLE) is a chronic autoimmune disease characterized by fluctuating symptoms, long-term medication use, bodily uncertainty, and complex self-management demands. These features can make patients’ experiences difficult to narrate, recognize, and integrate into everyday life. This study aimed to explore how Chinese women living with SLE use visual narratives to make sense of illness disruption, treatment burden, identity changes, and relational experience. **Methods**: This qualitative study used Virtual Photovoice, an online visual method in which participants generate and discuss photographs about lived experience, with eight Chinese women living with SLE. Data included participant-generated photographs, brief captions, SHOWeD-based written reflections structured around prompts that move from image description to broader reflection, and transcripts from three online Photovoice workshops. The data were analyzed using reflexive thematic analysis within a participatory-informed Virtual Photovoice design, informed by illness narrative theory. **Results**: Four themes were developed: Invisible Battlefield, Masks and Boundaries, Anchors of Order, and Longing to Be Seen. Participants used photographs and accompanying accounts to give form to fatigue, pain, and bodily uncertainty; negotiate the boundaries between concealment and disclosure; transform medication routines, dietary practices, and illness-related objects into anchors of order and agency; and contrast embodied relational support with clinical encounters experienced as distant or indicator-centered. **Conclusions**: The findings show how visual illness narratives can support meaning-making, self-recognition, and reflection on patient-centered communication among women living with SLE. Virtual Photovoice offers a narrative and participatory-informed approach for understanding psychological, embodied, and relational dimensions of chronic illness that are often difficult to express through routine clinical or everyday language.

## 1. Introduction

Systemic lupus erythematosus (SLE) is a chronic autoimmune disease that is often simplified in public discourse as “the butterfly rash on the face” (D’Cruz, 2006). This visible and emblematic sign, however, obscures the wider burden of the illness. Many people with SLE experience disabling fatigue, cognitive impairment, unpredictable systemic pain, and fluctuating disease activity, while still appearing outwardly healthy (Lupus Foundation of America, 2022; Salloum & Niewold, 2021). In China, this burden is not marginal: recent population-based and hospital-based studies have documented the continuing epidemiological and clinical significance of SLE, particularly among women (Li et al., 2024; Tan et al., 2021). Yet the significance of SLE cannot be understood only through prevalence, hospitalization, or clinical manifestations. The course of SLE varies widely across patients and over time, complicating diagnosis, treatment, and long-term self-management (Schattner et al., 2008; Pons-Estel et al., 2017). This gap between outward appearance and embodied experience makes SLE difficult not only to diagnose and manage, but also to narrate, validate, and communicate in everyday and clinical contexts.

The mismatch between bodily experience and outward appearance is not only a diagnostic or biomedical issue; it also shapes patients’ everyday self-management, social relationships, identity work, and interactions with healthcare providers. When patients’ embodied experiences conflict with their seemingly normal appearance, they may struggle to obtain social recognition and medical credibility (Poole et al., 2018). Many individuals with lupus report that their pain and fatigue are not fully witnessed or believed by family members, friends, or healthcare professionals, producing stigma, isolation, and psychological distress (Ghosh et al., 2021). Patients also often struggle to describe forms of exhaustion, brain fog, or pain that are difficult to verbalize, which can leave important dimensions of their lived experience misunderstood or marginalized in everyday and clinical discourse (Zartaloudi & Lazaras, 2021).

Although biomedical research on SLE has progressed, less attention has been paid to how patients narrate the psychological, relational, and practical work of living with the disease over time (Kalla, 2025). Existing studies relying on interviews and surveys have provided important insights, but they may not fully capture how embodied sensations, treatment routines, identity changes, and interpersonal experiences are organized into personally meaningful illness accounts (Huangfu, 2020). Illness narrative scholarship has shown that serious illness can disrupt coherence, identity, and self-understanding, requiring individuals to develop new meaning-making resources (Frank, 1995; Kleinman, 1988; Bury, 1982; Hydén, 2008). For SLE, this narrative work is complicated by fluctuating symptoms, uncertain disease trajectories, and the frequent mismatch between clinical indicators and lived experience. Understanding how patients construct and share illness narratives is therefore important for health psychology, because such narratives shape self-management, illness identity, support-seeking, and patient-centered communication.

### 1.1. Psychosocial and Communicative Challenges of SLE

Most studies on patients with SLE focus on the physical and psychological burdens of the illness and adaptation after diagnosis. However, SLE is also a deeply social experience (Mok, 2019). Unlike visible disabilities, many core symptoms of lupus, including chronic fatigue, joint pain, muscle soreness, headaches, and mood instability, are primarily subjective and difficult for others to observe. This invisibility is a structural feature of the illness experience rather than an incidental symptom (Wendell, 2001).

Invisibility generates uncertainty in patients’ daily lives and care trajectories. As Mishel (1988) argued, uncertainty in illness arises when individuals cannot construct a clear understanding of their disease state. For patients with SLE, unpredictable cycles of flare and remission make it difficult to interpret bodily changes and plan everyday life (Neville et al., 2021). This uncertainty extends beyond the medical domain, shaping social role performance, emotional well-being, and continuity of self-identity.

Such uncertainty can also become a source of stigma. When a patient’s outward appearance contrasts with their inner experience of pain or exhaustion, the credibility of their illness may be questioned (Poole et al., 2018). Patients may therefore engage in costly forms of impression management, such as hiding symptoms or performing normalcy, which can temporarily reduce suspicion but may also intensify isolation and emotional exhaustion (Ghosh et al., 2021). These dynamics affect not only social support but also chronic care. Family members and friends may struggle to respond appropriately to suffering they cannot see, while clinical encounters may prioritize laboratory indicators over fatigue, quality of life, or patient-reported concerns (Kattari & Beltrán, 2021; Zartaloudi & Lazaras, 2021).

### 1.2. Illness Narrative Theory and Meaning-Making in Chronic Illness

Illness narrative theory provides a useful lens for understanding how patients make sense of disruption and seek coherence amid chronic illness (Kleinman, 1988; Bury, 1982; Hydén, 2008). Kleinman (1988) emphasized that illness narratives reveal how suffering is interpreted and communicated within social and medical contexts. Bury (1982) similarly described chronic illness as a “biographical disruption” that forces individuals to reexamine self-concept and life trajectory. For Hydén (2008), telling the story of illness connects past and present while enabling patients to imagine possible futures.

Frank’s (1995) typology of illness narratives further illuminates how patients respond to serious illness. The restitution narrative treats illness as a temporary deviation from normal life, organized around recovery and return to normal. The chaos narrative describes suffering that lacks order, hope, or narrative coherence. The quest narrative frames illness as a reworked experience through which patients may develop new ways of being, valuing, and acting. These narrative forms are not mutually exclusive; they may coexist and shift across time and context (Brown et al., 2017). For individuals with SLE, whose symptoms fluctuate and are not always acknowledged by others, constructing a coherent illness narrative can be especially challenging. These difficulties highlight the need for participatory and expressive methods that allow patients to connect bodily sensations, treatment routines, emotional responses, and relational experiences into shareable accounts of chronic illness.

### 1.3. Photovoice as a Participatory Method for Visual Illness Narratives

Photovoice offers a methodological response to these challenges. Developed by Wang and Burris (1997) within participatory action research and empowerment theory, Photovoice is a participatory visual method that enables participants to document, reflect on, and discuss their lived realities through photography. Rather than positioning patients as passive subjects, it allows them to become active interpreters and co-producers of knowledge. Virtual Photovoice adapts this process to an online setting, allowing participants to share and discuss photographs through digital communication when in-person participation is difficult.

In health research, Photovoice has been used to explore experiences marginalized by mainstream discourse and difficult to express verbally (Catalani & Minkler, 2010). When language is inadequate to convey chronic pain, psychological distress, bodily change, or emotionally charged illness experiences, images can provide a more direct and safer medium of expression (Hergenrather et al., 2009; Latz et al., 2021; Smith et al., 2020). For narrative health psychology, this approach is especially valuable because it can reveal how patients use images, metaphors, objects, and dialogue to make sense of symptoms, treatment routines, self-management, and relational experience in everyday life.

The relevance of Photovoice for this study can be understood in three ways. First, by transferring the power of observation to patients, it supports patient-centered knowledge production and empowerment (Wang & Burris, 1997). Second, it provides visual anchors for intangible symptoms of SLE, such as fatigue, pain, and brain fog, allowing participants to narrate experiences that may be difficult to express through clinical or everyday language. Third, through structured dialogue frameworks such as SHOWeD, a reflective questioning technique that asks participants what they see, what is happening, how the situation relates to their lives, why it exists, and what can be done, Photovoice supports reflection on the personal, relational, and psychological meanings of illness (Wallerstein & Bernstein, 1988). In this way, Virtual Photovoice can be understood as a method for generating visual illness narratives: accounts in which images, captions, written reflections, and group dialogue together help patients organize bodily disruption, self-management, identity work, and relational experience into communicable forms.

### 1.4. Research Questions

This study uses Virtual Photovoice to examine how Chinese women living with SLE construct visual illness narratives in the context of a fluctuating chronic illness. By analyzing participant-generated photographs, captions, written reflections, and workshop discussions, the study explores how participants give form to difficult-to-verbalize symptoms, organize illness disruption, and connect self-management, identity work, and relational experience into meaningful accounts of living with SLE. Specifically, the study addresses the following research questions:

RQ1: How do Chinese women living with SLE use photographs and accompanying accounts to narrate bodily uncertainty, difficult-to-verbalize symptoms, and illness disruption?

RQ2: How do these visual illness narratives represent processes of self-management, identity negotiation, and relational recognition in everyday life?

RQ3: How can Virtual Photovoice contribute to meaning-making and patient-centered communication in the context of chronic illness?

## 2. Materials and Methods

### 2.1. Research Design

This study adopted a participatory-informed qualitative design grounded in Virtual Photovoice. The study was informed by illness narrative theory and treated participant-generated photographs, captions, written reflections, and workshop discussions as multimodal illness narratives. Rather than using narrative theory as a formal structural coding scheme, the analysis drew on it to attend to how participants organized bodily disruption, uncertainty, treatment routines, identity changes, and relational experience into meaningful accounts of living with SLE.

Virtual Photovoice was used because it allowed participants to create visual materials, attach personal meanings to those materials, and discuss illness experience in a shared online setting (Wang & Burris, 1997; Catalani & Minkler, 2010). Participants contributed through photograph production, caption writing, SHOWeD-based reflection, workshop dialogue, and consent-based image sharing. The research questions, formal coding, theme development, and final analytic synthesis remained researcher-led. For this reason, we describe the design as participatory-informed rather than as a full participatory action research project.

The study received ethical approval from the Research Ethics Committee of the Faculty of Humanities and Arts at Macau University (MUST-FA-2025018). It was conducted entirely online between November and December 2025 due to participants’ health conditions and geographical dispersion (Oliffe et al., 2023). The process included participant recruitment, image creation, written reflection, online Photovoice workshops, thematic analysis, and a small public exhibition of selected photographs and descriptions with participants’ consent.

### 2.2. Participants

Eight women living with SLE, aged 23 to 48, participated in this study. This sample reflects the gender disparity in SLE, which predominantly affects women of reproductive age, with a female-to-male ratio of approximately 9:1 (Lupus Foundation of America, 2022). Participants were recruited through Xiaohongshu, a popular social media platform in China. Recruitment used purposive convenience sampling. The first author posted a recruitment notice on Xiaohongshu, and interested individuals contacted the research team privately. The first author then provided study information and confirmed eligibility according to the inclusion criteria.

Inclusion criteria were (1) confirmed SLE diagnosis for more than one year; (2) current disease stability without hospitalization for disease flare or relapse; (3) willingness to engage in photography and written reflection; (4) ability to use smartphones and online meeting tools; and (5) ability to participate in in-depth Mandarin discussion. Pseudonyms were used to protect anonymity.

Participants came from diverse social backgrounds, including students, freelancers, and office workers. Some were newly diagnosed, while others had lived with SLE for more than a decade. Table 1 summarizes the participant information collected for this study. Despite these differences, all described managing invisible symptoms, treatment routines, and the social consequences of chronic illness.

### 2.3. Data Collection: Virtual Photovoice Workshops

The Virtual Photovoice process followed Wang and Burris’s (1997) three-step framework and was adapted to generate visual illness narratives through image creation, written reflection, and collective discussion. Participants were first invited to photograph their experiences of living with SLE. The instructions were open-ended, allowing participants to photograph anything that symbolized fatigue, pain, bodily change, medication routines, self-management, relationships, or daily life. Across the study, participants submitted 29 photographs, each accompanied by a brief caption. Asking participants to produce several photographs before choosing one focal image allowed them to consider different aspects of everyday illness experience before identifying the image they regarded as most meaningful.

Participants then selected one photograph for deeper reflection using the SHOWeD framework (Wallerstein & Bernstein, 1988): What do you See here? What is really Happening? How does this relate to Our lives? Why does this situation exist? What can we Do about it? This process produced eight focal photographs and eight SHOWeD-based written reflections. These focal materials became the basis for the online workshop discussions and subsequent thematic analysis.

Three online workshops were held via Tencent Meeting. A flexible workshop guide was used to support discussion flow while preserving participant-led elaboration. The guide organized the workshop around photograph sharing, personal interpretation, group discussion, shared or divergent experiences, self-management practices, and care-related concerns. The guide is provided in Appendix A. Sessions were recorded and transcribed with consent. The workshops generated approximately 6 h and 40 min of recordings and approximately 19,600 Chinese characters of transcript. All participants attended the three workshops, although some arrived late or left early in individual sessions. A more detailed overview of the Photovoice procedure and data corpus is provided in Appendix B.

After data collection, the eight participant-selected photographs and descriptions were displayed in a small campus exhibition in China, with participants’ consent, to raise awareness of SLE experiences and care needs. The exhibition was treated primarily as dissemination rather than as an additional data-collection stage.

### 2.4. Data Analysis

The analysis was conducted through reflexive thematic analysis (RTA) within a participatory-informed Photovoice design. Illness narrative theory informed the interpretation of the visual and verbal materials as multimodal accounts of chronic illness, while RTA guided the development of themes as analytic interpretations rather than descriptive topic summaries (Braun & Clarke, 2006, 2021b, 2024). The analysis followed a contextualist orientation, attending both to participants’ own meaning-making and to the broader interpretive patterns developed by the research team.

The eight focal photographs were not analyzed as isolated visual objects. Each photograph was treated as part of a linked analytic unit that included the image, its caption, the participant’s SHOWeD-based written reflection, and relevant workshop discussion. Visual features such as objects, bodily positioning, spatial arrangement, concealment, exposure, and metaphorical composition were noted in analytic memos. However, visual interpretation was anchored primarily in participants’ own explanations. When a photograph appeared to invite one reading but the participant explained it differently, the participant’s account was treated as the primary interpretive anchor, and the difference was recorded as an analytic tension.

Workshop discussions were analyzed as dialogic data. They allowed participants to elaborate on their photographs, compare experiences, affirm or complicate one another’s accounts, and introduce contextual details that were not always available in the captions or written reflections alone. These discussions informed coding by showing how individual visual narratives became connected to shared concerns about bodily uncertainty, visibility, treatment routines, support, and clinical communication.

Following Braun and Clarke’s reflexive approach, the first author conducted an iterative, inductive RTA across the full dataset, using NVivo 15 (Lumivero, LLC, Denver, Colorado, USA) for organization. Analysis involved repeated reading, viewing, and memoing; generating initial codes from participants’ accounts, including in vivo codes and articulated visual metaphors (Braun & Clarke, 2021b); developing candidate themes across cases and data types; refining themes through attention to coherence, distinctiveness, counter-examples, and alternative interpretations; and defining themes as analytic claims. Coding attended to both semantic and latent meanings, including how images and accounts made symptoms visible, negotiated stigma, expressed care needs, and supported self-definition in chronic illness management (Braun & Clarke, 2023, 2024).

### 2.5. Ethical Considerations

Ethical integrity was prioritized throughout. All participants provided informed consent after being informed of the study aims, procedures, voluntary nature of participation, and potential emotional risks. Participants could withdraw at any time without consequence. Because photographs could include personal or domestic spaces, participants controlled what they photographed, shared, and allowed to be displayed. Identifiable features, such as faces or specific locations, were blurred or excluded.

The research team also recognized the emotional vulnerability involved in reflecting on chronic illness. During workshops, participants could pause or withdraw from discussion if uncomfortable. After each session, debriefing emails were sent with mental health resources and support contacts. The study followed participatory research principles of respect, reciprocity, and empowerment while minimizing emotional and privacy-related risks.

### 2.6. Reflexivity and Analytic Rigor

Consistent with RTA’s emphasis on transparency and reflexive openness (Braun & Clarke, 2021a, 2024), researcher positionality was addressed throughout the analysis. As a female student researcher without personal experience of SLE, the first author shared a gendered and cultural context with participants while remaining an outsider to the embodied realities of chronic, invisible illness. Reflexive memoing was used to document assumptions, emotional responses, and key interpretive decisions during the analytic process.

A central reflexive issue concerned how participants’ photographs and accounts should be interpreted. Early analytic notes tended to frame several images primarily through vulnerability and victimization. Participants’ own workshop explanations complicated this reading. Their accounts redirected the analysis toward agency, everyday meaning-making, routine, and illness management, without denying suffering or bodily disruption. This reflexive shift was important in developing themes that captured both constraint and agency.

Rather than relying on inter-coder reliability, analytic rigor was supported through sustained engagement with the multimodal dataset, systematic memoing, team discussion, attention to divergent cases, and careful comparison across photographs, captions, written reflections, and workshop transcripts. Participant input was not treated as “validation” of themes in a positivist sense. Instead, participants’ explanations strengthened interpretive resonance and ethical accountability by helping the research team avoid imposing an overly vulnerability-centered reading. The final themes therefore reflect researcher-led analytic synthesis while remaining responsive to participants’ own accounts of their photographs and illness experiences.

## 3. Results

Reflexive thematic analysis of participants’ photographs, captions, SHOWeD-based reflections, and workshop discussions generated four themes: Invisible Battlefield, Masks and Boundaries, Anchors of Order, and Longing to Be Seen. Together, these themes form a visual narrative arc of living with SLE: from disrupted embodiment and difficult-to-verbalize symptoms, to negotiated visibility and social boundaries, to the reconstruction of order through treatment and daily routines, and finally to the desire for recognition within relational and clinical encounters. The workshop discussions contributed a different kind of material from the individual photographs and written reflections. Whereas photographs and SHOWeD responses foregrounded participants’ selected images and personal interpretations, the workshops generated dialogic material through comparison, elaboration, mutual recognition, and occasional disagreement. Affirming or sympathetic exchanges appeared in these discussions, but they did not form a separate theme; instead, they showed how participants collectively recognized and extended one another’s accounts across the four themes. Rather than representing isolated topics, the themes show how participants used images and accompanying accounts to connect bodily uncertainty, self-management, identity work, and relational experience into communicable illness narratives.

Although Section 3 presents selected photographs as illustrative cases, each theme was developed across the multimodal dataset rather than from a single participant account. We did not identify a case that fundamentally overturned the four-theme structure. However, several cases and interpretive moments complicated it: visibility was not always experienced only as unwanted exposure; treatment objects were not only burdensome but could also become evidence of endurance; and some photographs initially appeared to suggest vulnerability while participants’ own explanations emphasized agency, routine, or self-protection. These tensions were retained analytically rather than treated as errors or excluded as outliers.

### 3.1. Theme 1: The Invisible Battlefield: Narrating Fatigue, Pain, and Bodily Uncertainty

This theme captures how participants used photographs to make bodily disruption communicable when ordinary language felt insufficient. Across the dataset, participants narrated fatigue, pain, bodily unpredictability, and treatment-related distress in different ways: some emphasized migratory pain or repeated treatment, some described exhaustion as a collapse of daily capacity, and others linked bodily uncertainty to changes in mobility, appearance, or routine. These accounts showed that SLE symptoms were not experienced only as physical sensations, but also as disruptions to time, work, self-worth, and ordinary life. Everyday expressions such as “tired” or “painful” often seemed too weak to convey the intensity, unpredictability, and emotional weight of their symptoms. As a result, participants used ordinary images as visual anchors through which bodily sensations could be given form, shared, and interpreted.

#### 3.1.1. Metaphors of Pain: Needles as a Narrative of Repeated Confrontation

**Figure 1:**
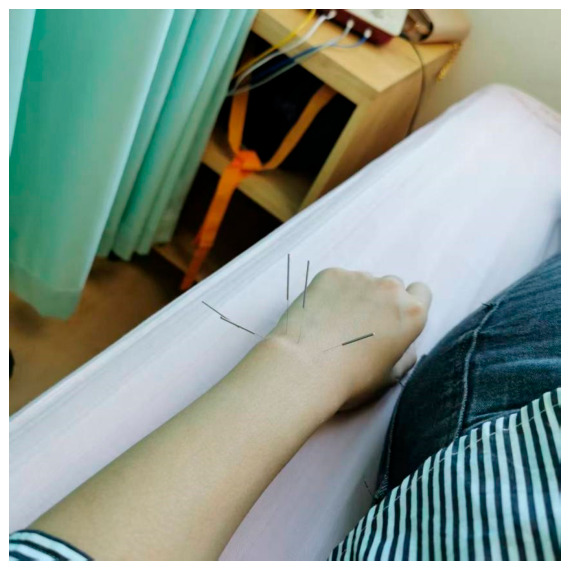
“Endless Needles,” photographer: Jia (pseudonym).

Jia, a 32-year-old participant who had lived with SLE for five years, used a photograph of acupuncture needles inserted into her hand and forearm to represent migratory pain and the repetitive burden of treatment (see Figure 1). She explained:

“*I consider myself lucky since I do not have those frightening rashes. But because my joints are affected, I often face migratory pain all over my body. I never know which part will hurt when I wake up the next day. I can only keep getting needling treatments, taking medicine, and applying patches. The image you see is my routine repeated countless times. After work, while others may go out for recreation, I can only go to the hospital alone for needling to gain temporary relief.*”

For Jia, the image communicated more than localized pain. The hand appeared passive and exposed, while the needles appeared active and invasive. Her description of never knowing “which part will hurt” conveyed the unpredictability of SLE pain and the loss of control it produced. The photograph also gave narrative structure to repetition: after work, time did not open into leisure or rest, but into another cycle of bodily repair. In this way, Jia’s image transformed a recurring and difficult-to-explain symptom into a shareable account of uncertainty, endurance, and interrupted everyday life.

#### 3.1.2. Metaphors of Fatigue: The Dead Battery and the Loss of Capacity

Fatigue was one of the most frequently described symptoms. Participants often emphasized that SLE-related fatigue was easily misunderstood as laziness or lack of motivation. Duoduo, a 25-year-old participant who had lived with SLE for two years, used the metaphor of a damaged battery to describe her experience:

“*Since getting sick, I can lie down for 20 h out of a 24-h day. I just want to lie down every day. I feel like a battery that can never be fully charged, one with a maximum capacity of only sixty percent. I feel like a useless dead battery.*”

**Figure 2:**
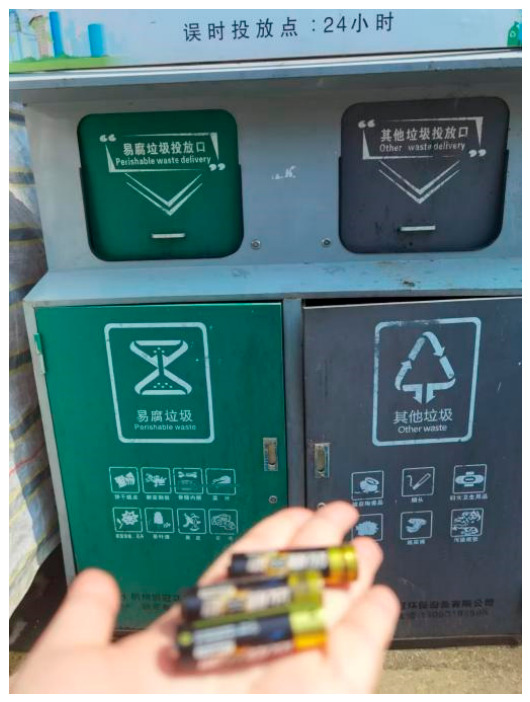
“Dead Battery,” photographer: Duoduo (pseudonym).

Duoduo’s image (see Figure 2) moved beyond the common metaphor of “low battery.” By photographing used batteries near a refuse station, she narrated fatigue as both functional depletion and a threatened sense of value. Her phrase “a maximum capacity of only sixty percent” suggested a changed bodily baseline rather than a temporary lack of energy, while “useless dead battery” conveyed the emotional consequences of no longer meeting expected levels of productivity. Across participants’ accounts, fatigue was not experienced merely as tiredness but as a symptom that reshaped self-worth, daily rhythm, and imagined possibilities of ordinary life.

### 3.2. Theme 2: Masks and Boundaries: Negotiating Visibility in Everyday Life

This theme examines how participants negotiated the unstable visibility of SLE in everyday life. Rather than appearing in one uniform form, visibility negotiation moved across three patterns in the dataset: concealing illness-related changes through objects such as wigs, masks, clothing, or selective disclosure; confronting situations in which illness became difficult to hide because of bodily change, mobility aids, or treatment side effects; and moving between concealment and disclosure depending on social context and anticipated responses from others. The photographs showed that visibility was not a fixed condition but a situated process shaped by bodily changes, material objects, social expectations, and the emotional cost of being seen or misunderstood.

#### 3.2.1. “My Gear”: Objects for Mediating Everyday Visibility

**Figure 3:**
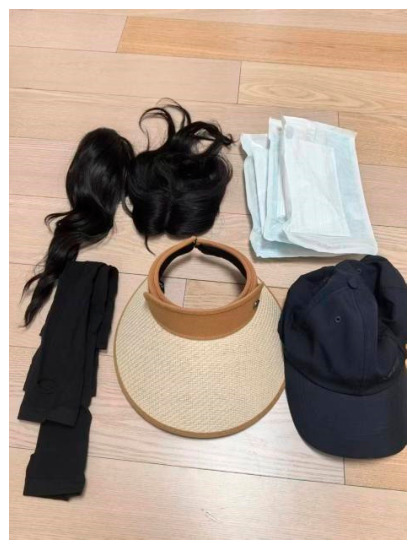
“My Gear,” photographer: Luo-yi (pseudonym).

Luo-yi, a 48-year-old participant with a 14-year illness course and lupus nephritis, presented a photograph of items (Figure 3) she described as her “going-out gear.” She had experienced total hair loss due to cyclophosphamide chemotherapy, as well as long-term side effects from steroids and kidney disease.

In Luo-yi’s account, these objects were not trivial accessories but material mediators between her changed body and the public gaze. They helped her decide how much of the illness would become visible in everyday encounters. She explained:

“*Good wigs matter a lot. If they look natural, I do not feel inferior. Cheaper ones are recognizable.*”

The photograph showed that everyday visibility was mediated through physical preparation, emotional effort, and financial cost. The wig helped Luo-yi feel less exposed and more able to enter ordinary social situations. At the same time, she emphasized the limits of such mediation. Even with a wig, steroid-induced facial changes remained difficult to conceal, especially because medical advice restricted the use of makeup. Masks and sun-protection items therefore carried multiple meanings: they protected her body, reduced unwanted attention, and allowed her to move through public space with a greater sense of control.

Other participants described similar uses of clothing, masks, cosmetics, or everyday objects to negotiate bodily visibility. However, they also described the strain of choosing between protecting health, preserving comfort, and limiting social exposure. This theme therefore captured not simply an effort to appear “normal,” but the ongoing work of deciding how much of illness could be shown, hidden, or translated through material objects.

#### 3.2.2. Wheelchairs and the Limits of Concealment

**Figure 4:**
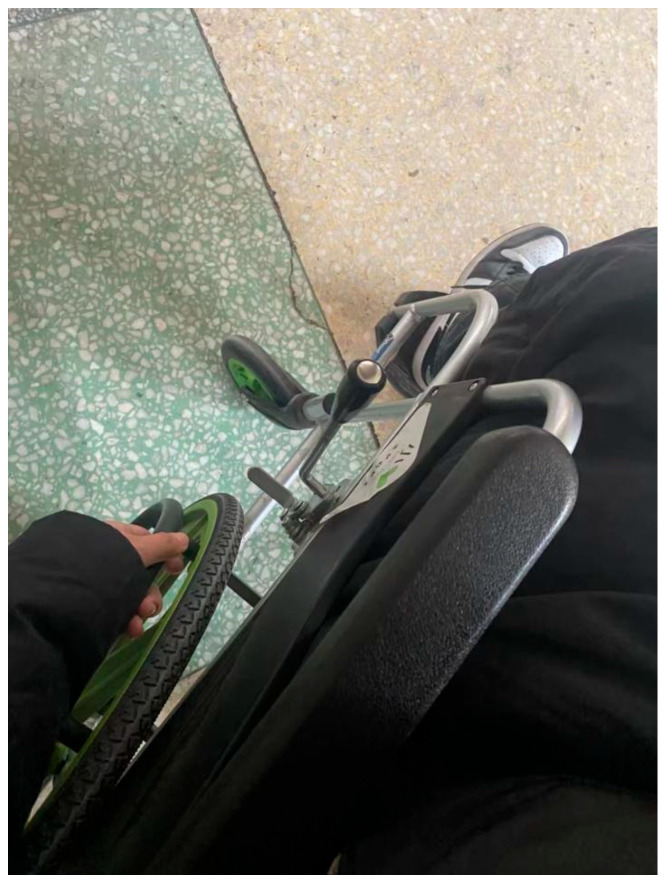
“My Wheelchair,” photographer: Xiao-zeng (pseudonym).

Xiao-zeng, a 26-year-old participant with a nine-year illness course and steroid-induced femoral head necrosis, photographed part of a wheelchair rather than herself (Figure 4).

In her explanation, the wheelchair represented reduced mobility and the difficulty of entering public and social spaces. Unlike Luo-yi’s “going-out gear,” the wheelchair could not be easily concealed, removed, or translated into ordinary appearance. Xiao-zeng explained that she “hardly goes out to socialize” because she relies on her father to carry her up and down stairs. For her, the wheelchair made illness visible in ways that were difficult to negotiate and intensified her sense of exposure in public.

When visibility could not be easily mediated, Xiao-zeng described reducing movement and limiting social participation. This was not only a response to physical pain but also a way to avoid being stared at, pitied, or treated as inconvenient. Together, Luo-yi’s and Xiao-zeng’s photographs show two related forms of visual illness narrative: one in which objects helped mediate bodily visibility and sustain participation, and another in which the limits of concealment led to withdrawal and the narrowing of everyday life.

### 3.3. Theme 3: Anchors of Order: Treatment Objects, Daily Routines, and Reworking the Self

This theme shows how participants used treatment objects and daily routines to reconstruct a sense of order within the uncertainty of SLE. Across cases, medication, injection materials, diet, rest, scheduling, and bodily monitoring appeared not simply as biomedical tasks, but as everyday practices through which participants managed disruption and reworked their sense of self. These routines were often ambivalent. They could be burdensome, repetitive, and body-altering, but they could also become sources of continuity, evidence of persistence, and practical forms of agency. The theme therefore does not treat treatment routines as mere compliance; it examines how participants narrated them as part of living with and making meaning from chronic illness.

#### 3.3.1. “My Evidence”: Medicine Boxes as a Material Narrative of Persistence

**Figure 5:**
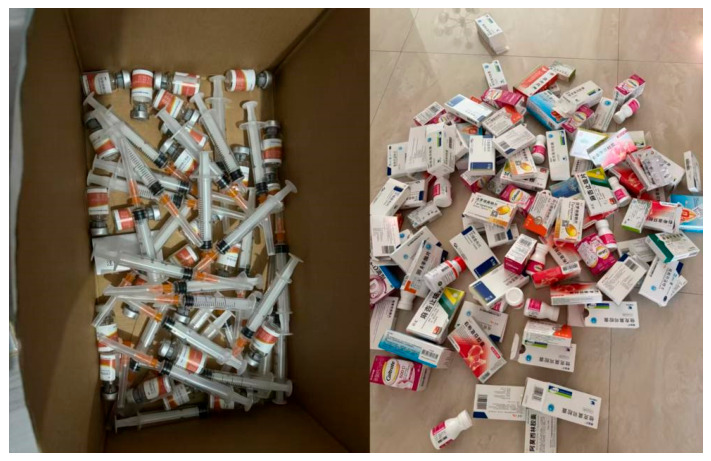
“My Evidence,” photographer: Juzi (pseudonym).

Juzi, a 30-year-old participant who had lived with SLE for eight years, presented a collage of empty medication boxes, bottles, and injection materials (Telitacicept) (Figure 5).

Juzi gave these objects new meaning:

“*I like collecting these boxes. I think they serve as evidence—proof that I’ve been persisting and fighting against lupus.*”

The image was analytically significant not simply because it displayed medication use, but because Juzi interpreted these “medical wastes” as proof of endurance. The collected boxes and vials made the repetitive work of treatment tangible and countable. Rather than treating medication remnants only as signs of dependence or bodily damage, Juzi reworked them into evidence that she had continued, endured, and acted.

Across the dataset, participants often described treatment as repetitive, exhausting, and difficult to explain to others. Juzi’s photograph showed one way of turning hidden treatment labor into a material narrative of persistence. It also illustrated how illness-related objects could support continuity and self-recognition in the ongoing process of living with SLE.

#### 3.3.2. “A Healthy Meal”: Daily Routine and Active Self-Management

**Figure 6:**
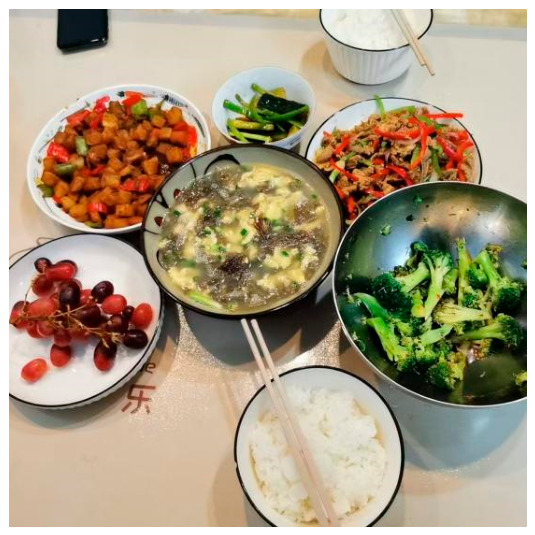
“A Healthy Meal,” photographer: Xiao-yi (pseudonym).

Xiao-yi, a 23-year-old participant diagnosed with SLE for more than one year, did not photograph medicines. Instead, she photographed a carefully prepared healthy meal (Figure 6).

In her explanation, the meal represented more than dietary compliance:

“*My doctor said being happy and eating well every day are very important for the illness. So I try to eat healthily every day. Since getting sick, my daily schedule and diet have completely changed.*”

Xiao-yi described dietary management not simply as restriction, but as an active and intentional practice through which she reorganized daily life after diagnosis. Rather than focusing only on prohibited foods, she emphasized choosing and preparing foods that supported a different way of living with illness.

Across cases, participants described self-management not only as following medical instructions but also as creating everyday routines that restored structure and control. Xiao-yi’s photograph captured this pattern clearly. By preparing meals with care, she was not only nourishing her body but also practicing self-care and continuity in daily life.

Together, Juzi’s and Xiao-yi’s photographs show two related ways participants turned treatment and routine into anchors of order: one by making the hidden labor of medication visible, and the other by transforming dietary practice into active self-management.

### 3.4. Theme 4: Longing to Be Seen: Relational Recognition and Clinical Disconnection

This theme focuses on participants’ desire for recognition within family, social, and clinical relationships. Across the dataset, recognition was described less as abstract sympathy than as practical and relational acknowledgment: being accompanied during treatment, having symptoms believed, receiving help with daily routines, or being allowed to describe bodily experience without immediate dismissal. Participants differed in where recognition was found. Some emphasized family members or partners, some referred to pets or cherished objects as sources of steadiness, and others focused more strongly on the gap between clinical indicators and their felt bodily experience. Together, these accounts show that participants wanted to be recognized not only as patients undergoing treatment, but as persons whose emotions, embodied knowledge, and everyday illness narratives mattered.

#### 3.4.1. “Mother’s Hands”: Embodied Presence and Steadfast Companionship

Across the workshops, participants repeatedly described close family members and partners as central to enduring illness, hospitalization, and uncertainty. What they valued most was not abstract encouragement but consistent presence, practical help, and emotional validation.

**Figure 7:**
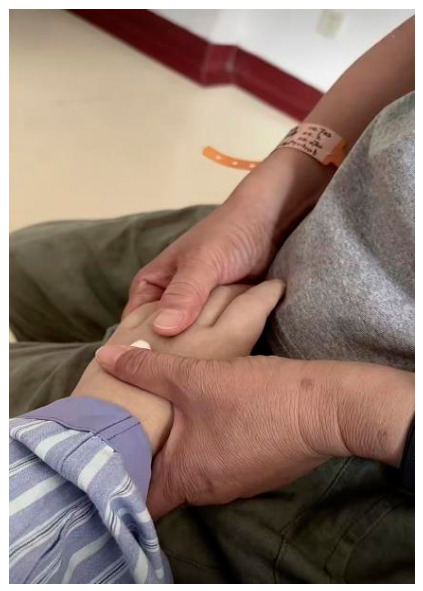
“Mother’s Hands,” photographer: Lucy (pseudonym).

Lucy, a 26-year-old participant who had lived with SLE for three years, took a photograph during a short admission for routine belimumab infusion. She showed her mother’s hands holding and pressing her own hand after an intravenous infusion (see Figure 7).

Lucy explained:

“*After diagnosis, my family’s support became really important. My mom stayed with me in the hospital, fetched medicine, went to the lab with me… She often said she felt heartbroken that she couldn’t take the pain for me. But I want to tell her that with her company, I truly found the belief to keep living.*”

In Lucy’s account, the photograph did not simply depict affection. It represented a form of companionship that helped sustain life under illness. The image showed support as bodily presence, practical action, and emotional steadiness rather than distant sympathy. Other participants also used images of parents, partners, pets, or cherished objects to represent relationships grounded in constancy and acceptance. These images suggested that dependable presence helped reduce the isolation of chronic illness and offered participants a relational basis for continuing their illness narratives.

#### 3.4.2. “I Hate You, ANA!”: Test Results and Indicator-Centered Misrecognition

**Figure 8:**
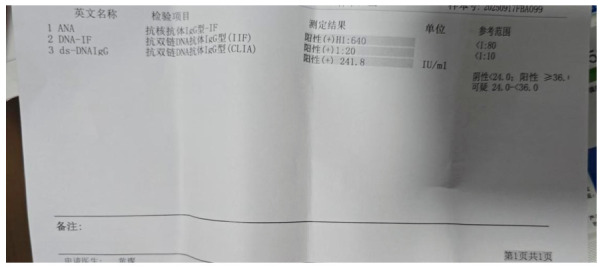
“I Hate You, ANA!” photographer: Xiao-mian (pseudonym).

In contrast to supportive relationships, participants also described medical encounters in which they felt unseen, unheard, or reduced to test results. Xiao-mian, a 27-year-old participant who had lived with SLE for 1.5 years, photographed her antinuclear antibody test report and titled it “I Hate You, ANA!” (Figure 8).

In Xiao-mian’s account, the report did not function as neutral medical information. It represented a recurring experience in which her embodied sense of feeling well was overridden by persistently abnormal laboratory indicators. Although she felt physically stable, these results led physicians to maintain or intensify treatment, which she experienced as frustrating and alienating.

Across cases, participants described similar moments in which their accounts of bodily experience seemed to carry less authority than biomedical evidence. The theme therefore captured a contrast between relational encounters that affirmed participants’ lived experience and clinical encounters that translated that experience into indicators. Participants did not reject biomedical monitoring; rather, they wanted their bodily knowledge, emotions, and everyday narratives of illness to be heard alongside laboratory evidence.

## 4. Discussion

This study used Virtual Photovoice to examine how Chinese women living with SLE constructed visual illness narratives in the context of a fluctuating chronic illness. The four themes show that participants did not simply document symptoms; they used photographs, captions, written reflections, and workshop dialogue to give form to bodily uncertainty, negotiate visibility in everyday life, create order through treatment and self-management routines, and articulate the need for recognition within relational and clinical encounters. Taken together, the findings suggest that living with SLE involves not only managing disease activity and treatment demands, but also narrating bodily disruption, sustaining self-recognition, and making lived experience communicable to others.

### 4.1. Visual Narratives of Bodily Uncertainty

The first theme showed how participants used visual metaphors to narrate pain, fatigue, and bodily uncertainty that were difficult to express verbally. This finding aligns with previous research showing that SLE symptoms are often fluctuating, difficult to communicate, and not always recognized by others (Poole et al., 2018; Zartaloudi & Lazaras, 2021). It also extends broader work on invisible chronic illness by showing how the absence of stable visible signs does not simply create problems of recognition but also generates a narrative problem: patients must find forms through which unstable and embodied sensations can be made shareable.

Participants’ images of needles and dead batteries provided concrete forms for experiences that ordinary language often failed to convey. This resonates with Frank’s (1995) account of chaos in illness experience, in which suffering can become fragmented and difficult to narrate. However, the present study shows that visual methods may offer an alternative route for organizing such disruption. Photovoice allowed participants to translate embodied uncertainty into images that could be shared, discussed, and interpreted collectively. This finding resonates with visual qualitative research on chronic pain and invisible illness, where participant-generated images have similarly been used to communicate embodied experiences that are difficult to express through words alone (Baker & Wang, 2006; Donnelly et al., 2021). This is important for narrative health psychology because symptoms such as fatigue, pain, and cognitive fog are not only functional burdens; they also shape self-understanding, emotional life, and patients’ capacity to produce coherent accounts of illness.

The findings also speak to Bury’s (1982) concept of biographical disruption. For participants, SLE disrupted not only bodily function but also time, work, social participation, and self-understanding. Jia’s repeated needling after work, for example, illustrated how chronic illness can reorganize daily life around cycles of symptom relief and bodily repair. Recognizing this disruption is essential for understanding SLE not only as a biomedical condition, but also as a narrative and psychological experience that exceeds laboratory results or disease activity scores.

### 4.2. Negotiating Visibility and Illness Identity

The second theme highlighted the social and psychological work involved in negotiating visible and less visible signs of illness. Participants described ongoing decisions about when to conceal bodily changes, when to disclose vulnerability, and when to withdraw from social situations. These findings extend previous studies showing that people with SLE often encounter disbelief, limited recognition, and social misunderstanding because their symptoms and bodily changes are not consistently legible to others (Ghosh et al., 2021; Poole et al., 2018).

Luo-yi’s “going-out gear” and Xiao-zeng’s wheelchair illustrate different points along a continuum of visibility negotiation. These findings can be understood in relation to Goffman’s (1986) account of impression management, in which individuals attempt to shape how they are perceived in social interaction. However, the present study adds that, in chronic illness, such negotiation is not simply a social performance; it is also tied to medical advice, bodily change, economic cost, material objects, and embodied vulnerability. For example, the tension between avoiding makeup for health reasons and wanting to enter public space without unwanted attention demonstrates how the body becomes narrated through objects, restrictions, and anticipated responses from others.

For health psychology, this finding suggests that visibility negotiation should not be treated as a peripheral social issue. It directly affects illness identity, self-management, social participation, emotional well-being, and patients’ willingness to seek or disclose support. Patient-centered communication therefore needs to address not only symptoms and treatment, but also the meanings patients attach to bodily change, public exposure, concealment, and withdrawal.

### 4.3. Treatment Routines, Self-Management, and Reworking the Self

The third theme showed that medication use, dietary routines, and illness-related objects were not experienced only as biomedical components of treatment. They also became part of participants’ efforts to create order, continuity, and agency in everyday life. This finding aligns with Huangfu’s (2020) observation that chronic disease can reshape the organization of daily life, and it extends this insight by showing how participants actively reinterpreted treatment routines and objects as meaningful resources for self-management.

Juzi’s collection of empty medication boxes made the hidden labor of treatment visible. Rather than representing medication only as dependency or bodily discipline, she treated the boxes as evidence of persistence. This finding can be read alongside Foucault’s (2002) discussion of the medical disciplining of the body and King’s (2004) feminist extension of bodily discipline; however, the participant’s own interpretation complicates a purely disciplinary reading. Medication routines were burdensome, but they could also be re-signified as evidence of endurance, agency, and continuity in an ongoing illness narrative.

Xiao-yi’s healthy meal similarly showed how self-management could become an everyday practice of agency rather than mere compliance. This is relevant to health psychology because living with SLE depends not only on medication adherence but also on lifestyle routines, emotional regulation, dietary practices, and patients’ ability to integrate illness into daily life. These findings suggest that self-management should be understood as both practical and meaning-making work. Supporting patients therefore requires attention to how they understand treatment, organize routines, and maintain a sense of self while living with chronic disease.

### 4.4. Recognition, Support, and Clinical Communication

The fourth theme revealed a contrast between relational encounters that affirmed participants’ lived experience and clinical encounters that could feel distancing or invalidating. Lucy’s photograph of her mother’s hands showed recognition as embodied presence, practical assistance, and emotional steadiness. This finding is consistent with research showing that family and social support are important for people living with chronic and difficult-to-recognize illness (Kattari & Beltrán, 2021).

By contrast, Xiao-mian’s test report illustrated a different kind of encounter: one in which the clinical meaning of laboratory results could override the participant’s felt sense of bodily stability. This does not mean that biomedical indicators are unimportant. Laboratory monitoring is essential in SLE care. The issue, rather, is communicative: when clinical encounters are organized mainly around indicators, patients may feel that their fatigue, pain, emotions, treatment burden, and everyday self-knowledge have little interpretive weight. Recent work on patient-reported outcome measures in SLE supports this point by showing that pain, fatigue, quality of life, and patient global assessment capture dimensions of disease burden that may not be fully represented by physician-assessed or laboratory indicators alone (Castrejón et al., 2024). Recent EULAR recommendations similarly emphasize comprehensive SLE management and non-pharmacological support, including education, self-management, health-related quality of life, and improved clinician–patient communication (Fanouriakis et al., 2024; Parodis et al., 2024).

This tension can be interpreted through Mishler’s distinction between the “voice of medicine” and the “voice of the lifeworld” (West & Mishler, 1986). Participants did not reject biomedical monitoring. Rather, they wanted clinicians to consider laboratory evidence together with their accounts of bodily sensations, emotional responses, and everyday illness management. The findings therefore support a communicative model in which clinical indicators and patient narratives are not treated as competing sources of evidence, but as complementary forms of knowledge.

### 4.5. Implications

This study offers several implications for narrative health psychology and patient-centered communication. First, patient-generated images can function as narrative prompts through which patients give form to fatigue, pain, cognitive difficulty, and bodily uncertainty, even when these experiences are not captured by outward appearance or laboratory indicators. Second, visibility negotiation should be understood as part of illness identity and psychological adjustment because decisions about concealment, disclosure, and withdrawal influence well-being, self-management, and social participation. This does not mean that healthcare providers should press patients to disclose sensitive experiences. Rather, clinical and support settings should create low-pressure opportunities for patients to talk about how illness visibility, concealment, or misunderstanding affects daily life when they wish to do so. Third, healthcare providers should ask not only whether patients follow treatment recommendations, but also whether treatment routines, diet, medication, rest, or bodily change have become difficult to integrate into everyday life. Such questions should be framed as optional, non-judgmental invitations rather than demands for disclosure. Finally, patient-centered communication should make space for patients’ own accounts of embodied experience alongside biomedical indicators. At the level of patient support programs and communication training, this means helping practitioners recognize patient narratives as complementary to clinical evidence, without shifting the burden of explanation entirely onto patients.

Virtual Photovoice may be useful not only as a research method but also as a reflective tool in narrative health psychology practice, patient support programs, and patient–provider communication training. By making hidden or difficult-to-verbalize aspects of illness shareable, images can help patients articulate concerns that may be difficult to express in routine clinical encounters. This potential aligns with the broader goals of Photovoice as a participatory and empowering method (Wang & Burris, 1997; Catalani & Minkler, 2010; Hergenrather et al., 2009), while also extending its relevance to visual illness narratives, chronic illness meaning-making, and patient-centered communication.

### 4.6. Limitations and Future Research

This study has several limitations. First, the sample was small and included eight Chinese women with SLE recruited through Xiaohongshu. Although this is appropriate for an in-depth qualitative Photovoice study focused on visual illness narratives, the findings should not be generalized to all people living with SLE. Future studies could include participants of different genders, ages, regions, socioeconomic backgrounds, and disease stages to explore how illness narratives may vary across social positions and disease trajectories.

Second, the study collected limited demographic information. To protect privacy and reduce identifiability in a small illness community, we collected age, illness duration, and occupation, but not education, income, geographic region, or rural/urban residence. This decision protected participants but limited our ability to analyze how socioeconomic position, regional context, or urban–rural differences shaped visual illness narratives. Future studies could collect broader demographic information when privacy risks can be adequately managed.

Third, the study was conducted entirely online. Virtual Photovoice made participation more accessible for individuals with health limitations and geographic dispersion (Oliffe et al., 2023), but it may also have shaped participation by excluding those with limited digital access or lower comfort with online communication. Future research could compare online, in-person, and hybrid Photovoice approaches to examine how different settings shape the production, sharing, and interpretation of visual illness narratives.

Fourth, the study centered on patient-generated visual illness narratives rather than triangulating them with clinician, family, or institutional perspectives. This was a methodological choice rather than only a limitation. Because the research questions focused on how women living with SLE made sense of their own illness experiences, the study privileged participant-generated photographs, captions, written reflections, and workshop dialogue as the primary sources of knowledge. Future research could ask different questions by examining how clinicians, family members, or patient organizations interpret and respond to patient-generated narratives.

## 5. Conclusions

Using Virtual Photovoice with eight Chinese women living with SLE, this study showed how participants constructed visual illness narratives through photographs, captions, written reflections, and workshop dialogue. These narratives gave form to bodily uncertainty, fatigue, pain, treatment burden, everyday visibility, self-management, and the desire for recognition in relational and clinical encounters. The findings contribute to narrative health psychology by showing that illness narratives are not only verbal accounts but may also be visual, material, embodied, and relational forms of meaning-making. Overall, Virtual Photovoice offers a participatory-informed and reflective approach for understanding how people living with SLE make their chronic illness experience communicable and psychologically meaningful. Rather than suggesting direct clinical transferability, the findings indicate how patient-generated images may serve as prompts for further research, patient support, and communication training.

## Figures and Tables

**Table 1:** Participant Characteristics (N = 8).

Pseudonym	Age	Illness Duration	Occupational Category
Jia	32	5 years	Sales
Duoduo	25	2 years	Student
Luo-yi	48	14 years	Unemployed
Xiao-zeng	26	9 years	Unemployed
Juzi	30	8 years	Education
Xiao-yi	23	1 year	Student
Lucy	26	3 years	Media
Xiao-mian	27	1.5 years	Freelancer

## Data Availability

Data available upon request due to restrictions (e.g., privacy, legal or ethical reasons).

## Abbreviations

The following abbreviations are used in this manuscript:

SLE	Systemic lupus erythematosus
RTA	Reflexive thematic analysis

## Appendix A

**Table A1:** Workshop Guide.

Workshop Component	Guiding Prompts	Purpose
Opening and orientation	Please briefly introduce the photograph you selected. Why did you choose this image for today’s discussion?	To help participants enter discussion through their own selected visual material.
Image description	What do you see in this photograph? What object, scene, body part, relationship, or moment does it show?	To begin with participant-led description rather than researcher interpretation.
Personal meaning	What is really happening in this image from your perspective? What does this photograph mean to you?	To connect the image to lived experience and personal interpretation.
Illness experience	How does this image relate to your experience of living with SLE? Does it connect to symptoms, treatment, uncertainty, daily routines, appearance, or relationships?	To explore how the photograph functions as a visual illness narrative.
Emotional and relational resonance	What emotions does this image carry for you? Do other participants see similar or different experiences in their own lives?	To support group dialogue, comparison, resonance, and divergence.
Self-management and care	Does this image say anything about how you manage illness, treatment, diet, rest, work, study, or relationships?	To connect visual narratives to everyday illness management.
Visibility and communication	Does this image relate to being seen, unseen, misunderstood, or recognized by others?	To explore invisibility, disclosure, stigma, and recognition.
Broader reflection	What do you wish family members, friends, clinicians, or the public could understand from this image?	To move from individual experience to broader social and care-related implications.
Closing	Is there anything about the photograph or your experience that has not yet been discussed?	To leave space for participant-led elaboration beyond the guide.

## Appendix B

**Table A2:** Virtual Photovoice Procedure and Data Corpus.

Stage	Participant Role	Researcher Role	Materials Generated or Used	Function in the Study
Recruitment and screening	Participants contacted the research team after seeing the study invitation and confirmed willingness to take part.	The research team screened participants according to eligibility criteria and provided study information.	Eligibility information including age, illness duration, occupation, SLE diagnosis duration, current disease stability, and ability to join online Mandarin discussion.	To identify eligible participants while minimizing collection of unnecessary personal information.
Photograph generation	Participants photographed experiences, objects, relationships, routines, or situations that represented living with SLE.	The research team provided open-ended instructions and ethical guidance on privacy and identifiable content.	29 submitted photographs in total; each photograph was accompanied by a brief caption.	To allow participants to document diverse aspects of everyday illness experience before selecting a focal image.
Focal image selection	Each participant selected one photograph she considered most meaningful.	The research team confirmed the selected image and prepared it for reflection and workshop discussion.	8 participant-selected focal photographs.	To center participants’ own judgment about which visual moment most strongly represented their illness experience.
SHOWeD-based written reflection	Participants wrote short reflections on their selected photograph using the SHOWeD prompts.	The research team provided the SHOWeD questions and clarified the task when needed.	8 SHOWeD-based written reflections.	To move from image description to personal, relational, and broader interpretation.
Online Photovoice workshops	Participants shared selected photographs and reflections, responded to one another, and discussed common experiences, visual metaphors, emotional resonances, self-management practices, and care-related concerns.	The researcher facilitated discussion using a semi-structured guide while allowing open-ended participant elaboration.	3 Tencent Meeting workshops; approximately 6 h and 40 min of recordings; approximately 25 transcript pages/19,600 Chinese characters. All participants attended all three workshops, although some arrived late or left early in individual sessions.	To generate dialogic data and examine how individual visual narratives connected to shared or divergent illness meanings.
Thematic analysis	Participants did not formally code data or approve final themes, but their workshop explanations shaped the interpretation of their photographs.	The first author led coding and theme development; the research team discussed analytic memos, candidate themes, divergent cases, and visual-verbal tensions.	Linked analytic units consisting of photograph, caption, SHOWeD reflection, and relevant workshop transcript excerpts.	To develop cross-case themes while keeping interpretation anchored in participants’ own accounts.
Exhibition and dissemination	Participants consented to the display of their selected photographs and descriptions.	The research team organized a small display of participant-approved materials.	8 selected photographs and descriptions displayed on a wall in Room 329, School of Journalism and Communication, Tsinghua University.	To raise awareness of SLE experiences and care needs. The exhibition was treated primarily as dissemination rather than as an independent data-collection stage.
